# FcγR-mediated antibody-dependent enhancement of SARS-CoV-2 infection in human monocyte-derived macrophages

**DOI:** 10.3389/fimmu.2026.1981260

**Published:** 2026-10-21

**Authors:** Marco Baratella, Francesca Sironi, Ilaria Marzinotto, Stefania Dispinseri, Andrea Canitano, Monica Tolazzi, Mauro Malnati, Marit J. van Gils, Vito Lampasona, Andrea Cara, Gabriella Scarlatti

**Affiliations:** 1 Viral Evolution and Transmission Unit, Institute of Immunology and Infectious Diseases, Istituto di Ricovero e Cura a Carattere Scientifico (I.R.C.C.S.) Ospedale San Raffaele, Milan, Italy; 2 Diabetes Research Institute, Istituto di Ricovero e Cura a Carattere Scientifico (I.R.C.C.S.) Ospedale San Raffaele, Milan, Italy; 3 National Center for Global Health, Istituto Superiore di Sanità, Rome, Italy; 4 Department of Medical Microbiology, Amsterdam University Medical Center (UMC), University of Amsterdam, Amsterdam, Netherlands

**Keywords:** ADE, antibody mediated enhancement of infection, coronavirus, Fcγ receptors, macrophages, SARS-CoV-2

## Abstract

**Introduction:**

Macrophages play a central role in innate immunity but can also facilitate viral dissemination through antibody dependent mechanisms, and, in turn drive the severity of the clinical symptomatology. Although macrophages are generally considered non permissive to SARS-CoV-2, anti-Spike antibodies may mediate virus cell interactions via Fc gamma receptors’ (FcγRs) binding, potentially enabling antibody-dependent enhancement (ADE) of infection. Whether anti-Spike antibodies in vivo can mediate FcγRs dependent SARS-CoV-2 infection in macrophages remains to be fully understood. Here we explored the capacity of SARS-CoV-2 Spike directed antibodies to mediate enhancement of viral infection in human monocyte derived macrophages (hMDMs) using an experimental system capable of discriminating between productive infection and passive viral uptake.

**Methods:**

SARS-CoV-2 entry and replication were assessed in hMDMs using Spike pseudotyped lentiviral vectors and infectious recombinant SARS-CoV-2 USA-WA1/2020 expressing enhanced green fluorescent protein (eGFP) or NanoLuc luciferase (nLuc). ADE was assessed in hMDMs using three human IgG1 anti-Spike monoclonal antibodies (mAbs): DH1047 and COVA1-16, neutralizing class 1 antibodies targeting the receptor binding domain (RBD), and DH1052, a non-neutralizing mAb targeting the N-terminal domain (NTD) as well as sera from five patients with severe COVID-19. An ACE2 negative FcγRII expressing B16-F10 cell line was used to confirm FcγR dependent infection and to screen a larger panel of patients’ sera. Viral entry and replication were quantified by luciferase activity, GFP fluorescence, nucleocapsid immunostaining, and infectivity assays of released virions.

**Results:**

Spike pseudotyped lentiviral particles were efficiently internalized by hMDMs but failed to establish infection, instead undergoing endo-lysosomal degradation. The infectious recombinant SARS-CoV-2 similarly failed to replicate in hMDMs unless preincubated with mAb DH1047, that mediated FcγR dependent infection in both hMDMs and FcγRII expressing B16-F10 cells. Sera from patients with severe COVID-19 did not promote ADE in hMDMs or FcγRII expressing cells at the experimental condition here used, and instead, dose dependently inhibited DH1047 mediated enhancement.

**Discussion:**

While hMDMs are intrinsically resistant to SARS-CoV-2 infection, productive replication can occur via FcγR mediated ADE in the presence of specific mAb and defined antibody to virus ratios. However, polyclonal antibody responses in patient sera suppress this mechanism, providing a potential explanation for the lack of convincing clinical evidence of ADE of infection in COVID-19 patients.

## Introduction

Among the key players in the innate immune response are macrophages, which function as frontline defenders by recognizing, engulfing, and eliminating pathogens (1). However, despite their protective role, macrophages can also act as reservoirs or carriers for certain viruses, contributing to viral persistence and dissemination. This dual nature has been documented in infections caused by various viruses, including human immunodeficiency virus (2, 3), human cytomegalovirus (4), and avian influenza viruses (5). In the context of severe acute respiratory syndrome coronavirus 2 (SARS-CoV-2), accumulating evidence suggests that human monocyte-derived macrophages (hMDMs) do not support productive viral replication (6–8). However, the absence of productive infection does not preclude a functional role for macrophages in shaping viral dissemination or immune pathology, particularly under conditions in which antibody-mediated pathways may alter virus–cell interactions. Antibody-dependent enhancement (ADE) of infection is a well-recognized immunopathological phenomenon in which virus-specific antibodies can facilitate viral entry into immune cells via Fc gamma receptors (FcγRs), potentially leading to enhanced viral replication and exacerbation of disease (9). While antiviral antibodies play a crucial role in neutralizing virions and blocking infection, some of these antibodies, under particular conditions, can augment viral uptake by FcγR-expressing cells such as monocytes, macrophages, and dendritic cells (10). This process occurs through the formation of virus and antibody immune complexes, which are internalized via FcγRs, potentially bypassing the requirement for canonical entry receptors. This phenomenon has been demonstrated in previous studies of several viral infections, including HIV (11), dengue (10), Ebola (12), Zika (13), and SARS-CoV-1 (14). In addition to enhancing viral replication within nonpermissive immune cells, ADE can also modulate cytokine production, thus contributing to hyperinflammatory responses (15). Concerning SARS-CoV-1, antibodies targeting the Spike envelope glycoprotein have been shown to mediate ADE by facilitating viral entry into FcγR-bearing cells, including B cells, monocytes, and macrophages (14). Whether a similar mechanism occurs with SARS-CoV-2 is not fully understood. There is limited but growing evidence that certain antibodies exhibit ADE properties *in vitro* under specific conditions (16, 17). Building on these observations, we selected three well-characterized anti-Spike monoclonal antibodies with defined binding sites and neutralization profiles to assess their capacity to mediate FcγR-dependent SARS-CoV-2 infection in hMDMs. To address this point, we established experimental systems capable of discriminating between true productive infection and passive viral uptake or phagocytosis and explored the susceptibility of hMDMs to SARS-CoV-2 infection and the potential of anti-SARS-CoV-2 antibodies to mediate ADE via FcγRs.

## Materials and methods

### Clinical and virological characteristics of the COVID-19 patients

A total of 13 coronavirus disease 2019 (COVID-19) patients with severe disease progression were selected from the COVID-19 cohort study COVID-BioB at the Istituto di Ricovero e Cura a Carattere Scientifico (I.R.C.C.S.) Ospedale San Raffaele (described in 18) (**Table 1**). Seven individuals were admitted to the intensive care unit (ICU) and survived, five were admitted to the ICU and died, and one was hospitalized for more than 7 days without ICU admission. The selected patients included three women and 10 men, with a mean age of 54.5 years ± 11.1 years. Serum samples from the patients were selected according to their neutralization titers and were collected between 5 and 32 days after symptom onset (average 11.4 days ± 7.7 days). Three patient serum samples exhibited high neutralization (ID_50_ > 25,000) capacity, six exhibited medium neutralization capacity (ID_50_ > 1,500; < 25,000), and four exhibited low or no neutralizing activity (ID_50_ < 1,500).

**Table 1:** Neutralizing antibody titers in sera from SARS-CoV-2-infected patients.

Patient ID	Gender	Age	Disease stages	Days from symptoms onset	Neutralization (ID_50_ against C3/Whuan 1/serum dilution)	Neuralization category
PZ_200201	M	48	Hospitalized > 7 days	10	61,533	High
PZ_200202	M	62	ICU alive	7	28,407	High
PZ_200133	M	61	ICU alive	7	26,629	High
PZ_200121	M	47	ICU alive	6	7,072	Medium
PZ_200141	F	65	ICU deceased	9	4,086	Medium
PZ_200178	M	55	ICU alive	32	6,378	Medium
PZ_200191	M	56	ICU deceased	7	18,791	Medium
PZ_200227	F	43	ICU deceased	5	15,454	Medium
PZ_200430	M	43	ICU alive	12	3,313	Medium
PZ_200471	M	75	ICU deceased	10	< 40	Low
PZ_200619	F	62	ICU deceased	8	463	Low
PZ_200677	M	58	ICU alive	23	1,248	Low
PZ_200814	M	34	ICU alive	12	508	Low

Neutralization titers are reported as ID_50_ values, defined as the reciprocal serum dilution required to achieve 50% inhibition of infection using a Spike C3 Wuhan pseudotyped lentiviral assay.

Patients are stratified according to clinical status (hospitalized or ICU, alive or deceased), demographic characteristics, and days from symptom onset at the time of sampling. Based on ID_50_ values, sera were categorized as high neutralizers (> 25,000), medium neutralizers (1,500–25,000), or low neutralizers (< 1,500).

### Lentiviral vector-based SARS-CoV-2 neutralization assay

Serum neutralization titers were determined using lentiviral vector (LV)-Luc/Spike-C3 pseudoviruses. Sera were heat-inactivated at 56°C for 30 min. LV-Luc/Spike-C3 pseudovirus-containing supernatant was incubated with six 3-fold serial dilutions of each heat-inactivated serum, starting at a dilution of 1:40, for 30 min at 37°C in 96-well plates. The virus-serum mixtures were then added in duplicate to VeroE6 cells seeded at a density of 20,000 cells/well. Plates were incubated at 37°C for 48 h. Subsequently, 100 μL of Bright-Glo reagent was added to each well, and the plates were incubated for 2 min at room temperature in the dark to allow cell lysis. Thereafter, 100 μL of cell lysate was transferred to a white 96-well plate, and relative light units were measured using a Mithras luminometer. The 50% inhibitory serum dilution was calculated using a linear interpolation method based on the mean of the duplicate responses. Neutralization was expressed as the reciprocal of the serum dilution giving 50% inhibition of relative light units RLUs) compared to the mean of the virus control wells. An ID_50_ below a 1:40 serum dilution was considered negative. Patients’ serum neutralization titers are available in Dispinseri et al. (18) with a more detailed description of the assays.

### Cell lines and preparation of hMDMs

VeroE6 cells (African green monkey kidney epithelial cells) and the B16-F10 and B16-F10 FcγRII (mouse epithelial-like cells) cell lines were cultured in Dulbecco’s modified Eagle’s medium (DMEM) with ultraglutamine, supplemented with 10% fetal bovine serum (EuroClone, Milan, Italy), 4-(2-hydroxyethyl)-1-piperazineethanesulfonic acid (HEPES) (1 M; EuroClone, Milan, Italy), 1% penicillin–streptomycin (EuroClone, Milan, Italy), 1% nonessential amino acids (NEAA) (EuroClone, Milan, Italy), and 1% sodium pyruvate (100 mM) (EuroClone, Milan, Italy). PBMCs were isolated from buffy coat preparations of healthy donors using Ficoll-HyPaque (EuroClone, Milan, Italy) density gradient centrifugation and further cultured to obtain hMDMs, as previously described (19). Briefly, PBMCs were resuspended in RPMI medium supplemented with 20% fetal bovine serum (EuroClone, Milan, Italy), 10% human serum (Sigma-Aldrich, St. Louis, MO, USA), HEPES (1 M; EuroClone, Milan, Italy), 1% penicillin–streptomycin (EuroClone, Italy), 1% NEAA (EuroClone, Milan, Italy), and 1% sodium pyruvate (100 mM) (EuroClone, Milan, Italy). PBMCs were cultured at a concentration of 1 × 10^6^ cells in 100 µL of RPMI in flat-bottom 96-well culture plates (Corning, NY, USA). After 5 days, nonadherent cells were removed by extensive washing with RPMI medium, and hMDMs were further cultured in DMEM supplemented with 20% fetal bovine serum (FBS) for 2 days (19) before incubation with the SARS-CoV-2 nLuc or enhanced green fluorescent protein (eGFP) viruses. All cell lines and hMDMs were maintained in a controlled environment at 37°C with 5% CO_2_ in humidified incubators.

### Flow cytometry

Expression of ACE-2, CD14, CD16 (FcγRIII), CD32 (FcγRII), and CD64 (FcγRI) on the cell surface of hMDMs, B16F10, and B16F10_FcRII was assessed. A total of 5 × 10^5^ cells were detached from the culture wells by incubation with Accutase for 30 min at 37 °C (for hMDMs) or Trypsin-EDTA 1×, 500 mg/L (Euroclone, Milan, Italy) for 1 min at 37 °C (for B16F10). The cells were then stained with a directly labeled RabMab anti-human Angiotensin-converting enzyme 2 (ACE2)_AF647 (Cat. No. MAB5676; Abcam, Cambridge, UK), mouse anti-human CD14_Pacific Blue (Cat. No. 558121; BD Pharmingen, San Diego, CA, USA), mouse anti-human CD16_APC-H7 (Cat. No. 560715; BD Pharmingen, San Diego, CA, USA), mouse anti-human CD32_FITC (Cat. No. 555448; BD Pharmingen, San Diego, CA, USA), and mouse anti-human CD64_PE (Cat. No. 558592; BD Pharmingen, San Diego, CA, USA) for 30 min at 4 °C. Thereafter, the cells were washed twice with cold phosphate-buffered saline (PBS) for 5 min, centrifuged at 1,300 rpm, and then fixed with 2% PFA for 10 min. The cells were subsequently acquired using a CytoFLEX LX (Beckman Coulter, Brea, CA, USA) flow cytometer and analyzed with FlowJo version 10.7.

### Pseudovirus production

Production of the lentiviral vector pseudotyped with the CT-truncated Spike glycoprotein from the Wuhan-Hu-1 strain (pSpike-C3) (20) and expressing luciferase used for the neutralization assay was accomplished as described (18). Briefly, 3.5 × 10^6^ 293 T Lenti-X cells (Clontech) were seeded in 10 cm Petri dishes (Corning Incorporated, Life Sciences, Oneonta, NY, USA), maintained in high-glucose Dulbecco’s modified Eagle’s medium (DMEM; 4.5 g/L) (Gibco, Life Technologies Italia, Monza, Italy) supplemented with 10% fetal calf serum (Corning, Mediatech Inc., Manassas, VA, USA) The cells were then transiently transfected with transfer vector plasmid pGAE-LucW, packaging plasmid pADSIV3+, and pseudotyping plasmid (pSpike-C3) using the JetPrime transfection kit (Polyplus Transfection, Illkirch, France), following the manufacturer’s recommendations and using a 1:2:1 ratio (transfer vector:packaging plasmid:pseudotyping Spike plasmid). At 48 h posttransfections, culture supernatants containing the Spike-pseudotyped lentiviral vector expressing luciferase (LV-Luc/Spike-C3) were collected, cleared of cellular debris by low-speed centrifugation, passed through a 0.45-μm pore-size filter unit (Millipore, Billerica, MA, USA), and stored in 1 mL aliquots at − 80°C until use.

Production of the GFP-labeled lentiviral vectors expressing mCherry as a reporter protein and pseudotyped with Spike from SARS-CoV-2 (LV-mCherry/S) or the control envelope G protein from the vesicular stomatitis virus (LV-mCherry/G) was accomplished as described (21, 22). Briefly, Lentiviral vectors were produced by transient transfection using the JetPrime transfection kit (Polyplus Transfection Illkirch, France) in 293 T Lenti-X cells as described above using transfer vector plasmid pGAE-mCherry, packaging plasmid pADSIV3+, pseudotyping plasmid (pSpike-C3 or phCMV-vesicular stomatitis virus G protein [VSV.G]), and plasmid pCDNA3-SIVGag-GFP, using a 1:2:1:0.5 ratio (transfer vector:packaging plasmid:pseudotyping plasmid:GFP plasmid). Plasmid pCDNA3-SIVGag-GFP, expressing the codon-optimized sequence of SIVGag fused to the GFP sequence (23), was added to the plasmid mixture during vector preparation to produce GFP-labeled virions and allow for microscopic visualization of LV-mCherry/S and LV-mCherry/G (22, 24). Supernatants containing the vectors were collected 48 h posttransfection, filtered through 0.45-µm filters (Millipore, Burlington, MA, USA), concentrated by ultracentrifugation, and stored at − 80 °C until use. Recovered LV-mCherry/G or LV-mCherry/S were titrated by the RT activity assay and by flow virometry analysis (22) and used for the transduction of hMDMs or VeroE6. A schematic representation of the expression cassettes of the plasmids described above is shown in **Supplementary Figure S1**.

### SARS-CoV-2 viruses

SARS-CoV-2 USA-WA1/2020 recombinant infectious clone with Nano-luciferase gene (SARS-CoV-2_nLuc) or with enhanced Green Fluorescent Protein (SARS-CoV-2_eGFP) was obtained from BEI Resources (NR-54002, NR-54003). For the expansion of SARS-CoV-2_nLuc and SARS-CoV-2_eGFP viruses, 8 × 10^6^ VeroE6 cells were seeded in a T75 cell culture flask and cultured in complete DMEM. After 24 h, the viruses were added at a MOI of 0.00006. After 3 days, 10 mL of culture supernatant was collected and titrated as follows. 5.0 × 10^5^ VeroE6 cells were seeded in a six-well plate in complete DMEM for 24 h, after which the medium was removed and replaced with 1 mL of DMEM supplemented with 1% FBS containing 10-fold serial dilutions of SARS-CoV-2_nLuc and SARS-CoV-2_eGFP, respectively. After 1 h of incubation, the viral inoculum was removed, and each well was overlaid with 1 mL of methylcellulose (Sigma-Aldrich, St. Louis, MO, USA) dissolved in DMEM supplemented with 5% FBS. After 3 days, the methylcellulose was removed, and the cells were fixed with 6% paraformaldehyde (Sigma-Aldrich, St. Louis, MO, USA) for 30 min at room temperature. Subsequently, the cells were stained with 1% crystal violet (Sigma-Aldrich, St. Louis, MO, USA) in 20% ethanol. Plaques were counted, and the virus titer was calculated in terms of plaque-forming units (PFU)/mL.

### Monoclonal antibodies

mAbs DH1047 and DH1052 were kindly provided by Dr. Kevin O Saunders and Dr. David C Montefiori. The variable regions of both antibodies were synthesized and cloned into a human IgG1 backbone, as described by Li et al. (16). mAb COVA1–16 was kindly provided by Dr. Marit J. van Gils (25). mAbs DH1047 and COVA1–16 are class I antibodies with neutralizing activity (16, 25), with half-maximal inhibitory concentrations (ID_50_) of 0.10 µg/mL and 2.45 µg/mL, respectively, measured using a Spike C3 Wuhan-pseudotyped lentivirus. DH1047 has also been reported to mediate ADE in ACE2-negative, FcγR-positive engineered TZM-bl cells through an FcγR–dependent mechanism. The third antibody, DH1052, targets the N-terminal domain (NTD) and is nonneutralizing, exhibiting FcγR-independent infection-enhancing activity in TZM-bl cells (16).

### Viral infection and luciferase assays

VeroE6, B16-F10, B16-F10_FcγRII, and hMDM cells were cultured in 96-well clear flat-bottom polystyrene TC-treated microplates (Corning, NY, USA). The culture medium was subsequently replaced with SARS-CoV-2_nLuc at different MOIs, either alone or preincubated for 1 h with varying concentrations of mAbs DH1047, DH1052, COVA1–16, or patients’ sera, diluted in 100 µL of DMEM. After 6 h, the viral input was removed, cells were washed extensively with PBS, and cultured in complete DMEM. NanoLuciferase detection was performed using the Nano-Glo Luciferase Assay System (Cat. No. N1120; Promega, Madison, WI, USA) according to the manufacturer’s instructions. Briefly, 40 µL of Nano-Glo substrate, diluted in an equal volume of DMEM, was added to each well. Following a 3-min incubation at room temperature, 50 µL of the suspension was transferred to 96-well half-area black flat-bottom plates (Cat. No. 3694, Corning, NY, USA), and luminescence readings were taken within 5 min using a Berthold Technologies TriStar 5 luminometer. In experiments with patient sera, a DH1047 concentration curve was added as a positive control to each plate.

### FcγRII receptor-blocking assay

For FcγRII blocking experiments, 10,000 B16-F10_FcγRII cells were seeded in 96-well flat-bottom microplates (Corning, NY, USA). Prior to infection, cells were pre-incubated with 0.4 µg/mL of an anti-FcγRII monoclonal antibody (mouse anti-human CD32-FITC, Cat. No. 555448; BD Pharmingen, San Diego, CA, USA) for 1 h at 37 °C. After receptor blocking, cells were washed three times and incubated with 100 µL of SARS-CoV-2_nLuc preincubated with 3 or 10 µg of mAb DH1047. After 6 h, the viral input was removed, cells were extensively washed with PBS, and cultured in complete DMEM. NanoLuc activity was measured after 24 h using the Nano-Glo Luciferase Assay System (Cat. No. N1120, Promega, Madison, WI, USA), according to the manufacturer’s instructions.

### Detection of viral replication by immunohistochemistry and immunofluorescence

VeroE6, B16-F10, B16-F10_FcγRII cells, and hMDMs were cultured in ibiTreat 18-well µ-Slide polymer coverslips (ibidi GmbH, Gräfelfing, Germany) in complete DMEM. For pseudovirus experiments using LV-mCherry/S or LV-mCherry/G, 3.5 × 10^6^ LV-mCherry/S or 2.5 × 10^6^ LV-mCherry/G pseudoviral particles were added to the culture medium for 24 or 48 h. For SARS-CoV-2 infection, the culture medium was replaced with different MOIs of SARS-CoV-2_eGFP, either alone or preincubated for 1 h with different concentrations of the mAbs DH1047, DH1052, or COVA1–16. After 6 h, the viral inoculum was removed, cells were washed extensively with PBS, and further cultured in complete DMEM. At the selected time points, cells were washed three times with PBS and fixed in 4% PFA for 2 h.

SARS-CoV-2 or pseudovirus infection was detected either by eGFP fluorescence or by immunostaining of the nucleocapsid protein (NP). The NP was detected with an anti-SARS-CoV-2 nucleocapsid antibody (Cat. No. GTX135357; GeneTex, Irvine, CA, USA) for 2 h at room temperature. After three PBS washes, a secondary Alexa Fluor™ 546 Goat anti-Rabbit IgG (H + L) antibody was added for 1 h at room temperature.

For pseudovirus cellular localization, early endosomes were stained using a Rabbit monoclonal anti-early endosome antigen 1 (EEA1) antibody (Abcam, Cambridge, UK, clone EPR4245, Cat. No. ab109110), followed by an Alexa Fluor™ 546 Goat anti-Rabbit IgG (H + L) secondary antibody. Lysosomal compartments were stained using LysoTracker™ Deep Red (Cat. No. L12492; Invitrogen, Thermo Fisher Scientific, Waltham, MA, USA). Nuclei were stained with Hoechst 33342 (Thermo Fisher Scientific, Waltham, MA, USA) at 1 µg/mL. Following staining, cells were washed twice with PBS (Euroclone, Milan, Italy). Imaging was performed using a GE Healthcare DeltaVision™ Ultra microscope equipped with either a × 20 U Plan FI Dry Ph1 objective or a × 60 Plan Apo (NA 1.42) oil immersion objective. Image analysis was conducted using SoftWorx and/or Fiji (ImageJ) v2.8.0.

### Liquid-phase luciferase immune precipitation system assay

Serial dilutions of mAb DH1047 and patients’ sera were used, and their binding strength to the SARS-CoV-2 Spike glycoprotein (S1 + S2 domains) was measured by the LIPS assay as previously described (18, 26). The binding raw data signal (measured as light units) of mAb DH1047 dilutions at known concentrations was compared to the binding signal of patients’ sera dilutions to enable the selection of the serum dilutions that exhibited relative binding comparable to 1 μg of DH1047, a concentration determined to consistently induce ADE in our experiments.

## Results

### Abortive transduction of Spike-pseudotyped lentiviral vector in hMDMs

To address whether hMDMs can be productively infected by SARS-CoV-2, we initially used a LV coding for the mCherry reporter protein, which was pseudotyped with either the SARS-CoV-2 Spike protein (LV-mCherry/S) or the broad tropism VSV.G (LV-mCherry/G). Both vectors were produced in the presence of a Gag-GFP fusion protein, which labels the pseudotyped LVs with GFP, to evaluate viral particle uptake regardless of mCherry expression. Spike-pseudotyped LV-mCherry/S were efficiently internalized by hMDMs. By 6 h postincubation (hpi), intracellular GFP-positive particles became detectable in a subset of cells, and at 24 h hpi all cells showed GFP positivity (**Figure 1**), indicating progressive particle internalization. However, mCherry-positive hMDMs were never detected at any time point, indicating that LV-mCherry/S transduction of hMDMs is abortive (**Figure 1A**). Similarly to hMDMs, all ACE2-positive VeroE6 cells were GFP-positive at 24 hpi, indicating robust lentiviral particle entry but, at variance with hMDMs, expressed increasingly mCherry by 48 hpi, confirming successful completion of a single replication cycle (**Figure 1B**). To confirm that the replication block was specific to Spike-pseudotyped LV-mCherry/S, hMDMs were infected with the VSV.G-pseudotyped LV-mCherry/G (**Figure 1A**). Transduced hMDMs showed robust GFP expression and clear mCherry positivity, confirming that hMDMs fully support lentiviral replication when entry occurred through the VSV.G receptor (27), suggesting that the postentry restriction observed with Spike-pseudotyped LV did not reflect an inability of hMDMs to sustain viral replication.

**Figure 1:**
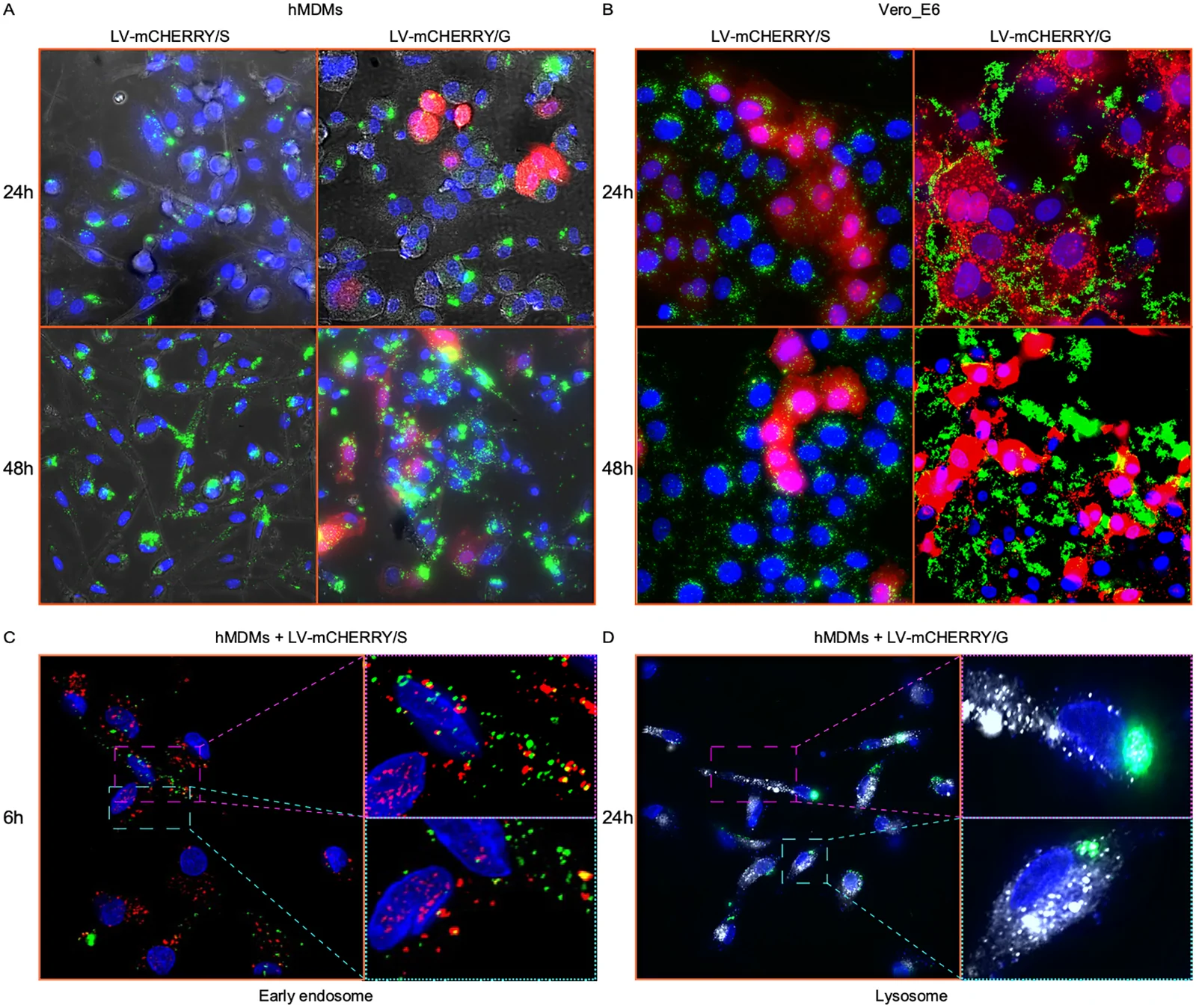
Cellular uptake, processing, and intracellular trafficking of LV-mCHERRY/S and LV-mCHERRY/G in hMDMs and VeroE6 cells. **(A)** Representative images of hMDMs exposed to 3.5 × 10^6^ LV-mCHERRY/S or 2.5 × 10^6^ LV-mCHERRY/G for 24 and 48 h. GFP (green) reports viral particle distribution, mCherry (red) indicates viral replication. Nuclei are counterstained with Hoechst (blue). HMDMs exposed to LV-mCHERRY/S showed no evidence of replication, despite consistent intracellular GFP signal, whereas exposure to LV-mCHERRY/G resulted in productive lentiviral replication, as indicated by the robust mCherry signal (red). **(B)** Vero_E6 cells, used as a positive control for infection, exhibited efficient viral entry, cytoplasmic accumulation of viral particles (green), and strong mCherry replication (red) signals following exposure to both LV-mCHERRY/S and LV-mCHERRY/G. **(C)** High-resolution imaging of hMDMs at 6 h postexposure shows early endosomal localization of GFP viral particles (green), demonstrated by colocalization (yellow) with the early endosome marker EEA1 (red). Insets highlight individual early endosomal compartments containing pseudotyped particles. **(D)** At 24 h postinfection, a subset of viral particles (green) localizes to lysosomes (white), as shown by colocalization with Lysotracker. Insets show lysosomal compartments enriched in viral structures. All images were acquired using a GE Healthcare DeltaVision microscope.

To define the intracellular fate of Spike-pseudotyped particles in hMDMs, imaging was performed at early and late time points after transduction with GFP-labeled LV-mCherry/S. At 6 hpi, GFP-labeled LV-mCherry/S showed colocalization with EEA1, indicating uptake into early endosomes (**Figure 1C**), while at 24 hpi, colocalization with LysoTracker demonstrated routing into lysosomal compartments (**Figure 1D**). No cytosolic signal compatible with productive replication was observed, consistent with lysosomal degradation rather than successful infection. Collectively, these results demonstrate that Spike-pseudotyped LVs entering hMDMs are subsequently routed towards endolysosomal degradation and cannot initiate a replication cycle. These findings prompted follow-up experiments using fully infectious SARS-CoV-2 to test whether virions can overcome the postentry block observed with Spike-pseudotyped LV particles.

### SARS-CoV-2 fails to replicate in hMDMs

HMDMs and VeroE6 cells, the latter used as a positive control for SARS-CoV-2 infection and replication, were incubated with serial dilutions of SARS-CoV-2_eGFP or SARS-CoV-2_nLuc. Both viruses replicated in VeroE6 cells (**Figures 2A, B**, left). Titration experiments showed a viral input-dependent GFP fluorescence signal as early as 6 hpi at both MOI 0.4 and 0.04, whereas at lower MOI (0.004 and 0.0004) the signal became detectable at 24 hpi (**Figure 2A**). Thereafter, the signal plateaued at 48 hpi and was followed by extensive cell death by 72 hpi in all experimental conditions. MOI 0.4 and 0.04 of SARS-CoV-2_nLuc showed a similar increase in RLU by 24 hpi in VeroE6 cells (1.2 and 1.5 × 10^7^ RLU/well, respectively) and plateaued thereafter (**Figure 2B**, left). Therefore, both MOI 0.4 and 0.04 were used to infect hMDMs. In contrast to VeroE6 cells, no increase in RLUs, no GFP signal, and no cytopathic effect were detected in hMDMs throughout the 14-day observation period, indicating an absence of productive viral replication (**Figures 2C, B**, right).

**Figure 2:**
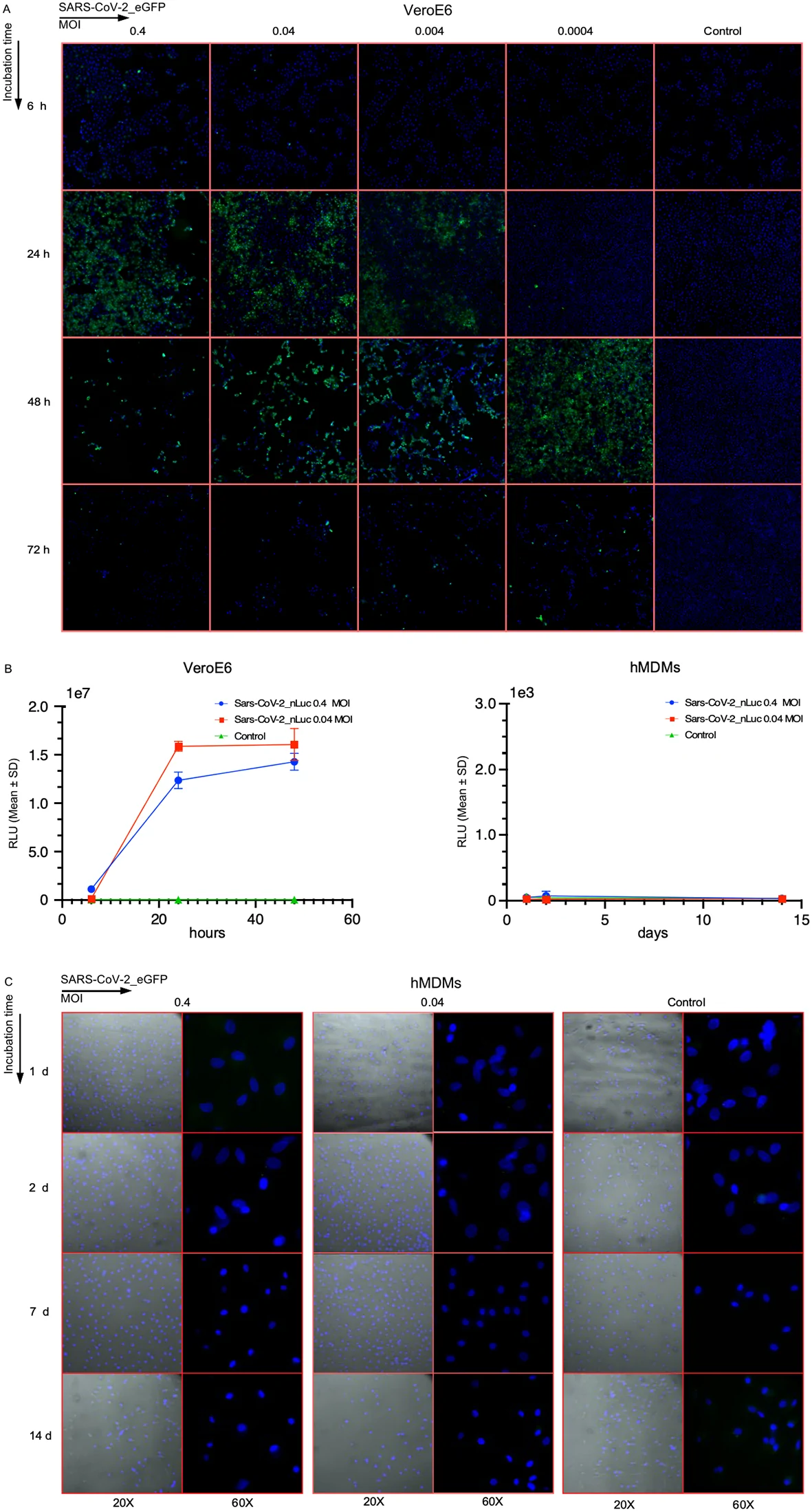
HMDMs are resistant to productive SARS-CoV-2 infection. **(A)** VeroE6 cells incubated with SARS-CoV-2_eGFP (MOIs 0.4, 0.04, 0.004, and 0.0004) displayed GFP fluorescence (green) as early as 6 hpi at the highest MOI. By 48 h, nearly all cells were infected, even at the lowest MOI. At 72 hpi, widespread cell death was observed, with surviving cells exhibiting irregular, apoptotic-like morphologies. **(B)** Productive infection was quantified by measuring RLU in VeroE6 cells and hMDMs infected with SARS-CoV-2_nLuc at MOIs of 0.4 and 0.04. VeroE6 cells showed a robust, time-dependent increase in RLU, consistent with active viral replication, whereas hMDMs incubated with SARS-CoV-2_nLuc for 1, 2, and 14 days displayed no increase in luciferase signal over time, indicating that hMDMs do not support productive SARS-CoV-2 replication. **(C)**Widefield microscopy images of hMDMs exposed to SARS-CoV-2_eGFP at MOIs of 0.4 and 0.04 for 1, 2, 7, and 14 days show no detectable GFP signal (green), confirming that hMDMs are not permissive to infection. Images were acquired using a GE Healthcare DeltaVision microscope with a U Plan FI × 20 dry objective or with a × 60 oil-immersion objective. Nuclei are shown in blue.

### DH1047 monoclonal antibody mediates SARS-CoV-2 enhancement of infection in an FcγRII-expressing cell line

To investigate the potential for ADE of SARS-CoV-2 infection, we utilized the murine epithelial ACE2-negative cell line B16–F10 and the genetically modified variant overexpressing human FcγRII (B16-F10_FcγRII) (**Supplementary Figure S2A**), which are resistant to SARS-CoV-2 infection (**Figure 3B**). Serial dilutions of the three distinct anti-Spike antibodies were preincubated with SARS-CoV-2_nLuc (MOI 0.04) prior to addition to both cell lines. After 24 h, the B16-F10_FcγRII cells showed viral replication only in the presence of mAb DH1047, with luciferase activity peaking at 1 μg/mL (~ 1,940 RLU/well) (**Figure 3A**). No infection was observed with mAbs DH1052 and COVA1–16 at any of the tested concentrations, indicating that enhancement of infection was specifically mediated by neutralizing mAb DH1047. Furthermore, to confirm that infection was specifically mediated by FcγRII, we performed Fc receptor-blocking experiments by pre-incubating the cells with an anti-FcγRII mAb prior to infection. FcγRII blockade abrogated infection, confirming the specific involvement of this receptor (**Supplementary Figure S3**). To further characterize viral infection dynamics, a complementary assay was conducted using SARS-CoV-2_eGFP. Consistent with the luciferase assay, B16-F10_FcγRII cells were infected as early as 24 hpi, with the proportion of GFP-positive cells correlating with DH1047 concentration and mirroring the luciferase signal distribution profile. No GFP signal was observed in parental B16-F10 cells at any time point (**Figure 3B**). This demonstrates that the DH1047 monoclonal antibody can mediate enhancement of SARS-CoV-2 infection in an FcγRII-expressing, ACE2-negative cell line, whereas mAbs DH1052 and COVA1–16 do not promote viral infection under the same conditions.

**Figure 3:**
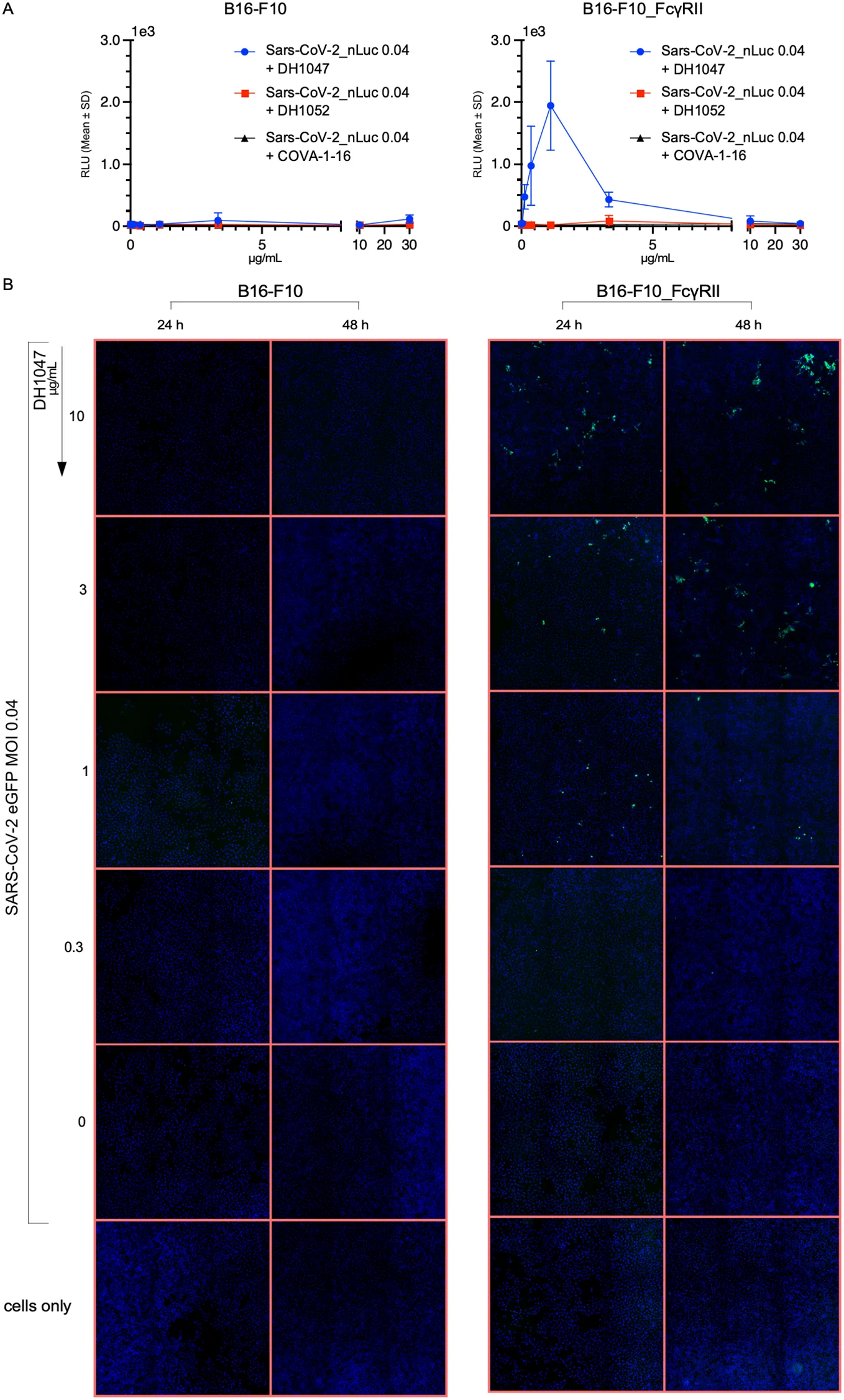
MAb DH1047 mediates ADE of SARS-CoV-2 infection via FcγRII in B16-F10 transgenic cells. **(A)** RLU values were measured in parental B16-F10 cells and B16-F10 cells expressing FcγRII (B16-F10_ FcγRII) after exposure to SARS-CoV-2 nLuc (MOI 0.04) for 24 h. Prior to incubation with the cells, the virus was pre-incubated for 1 h with a threefold serial dilution of DH1047, DH1052, or COVA1–16 mAbs (starting at 30 μg/mL). A significant increase in RLU was observed only in FcγRII-expressing cells incubated with the DH1047-SARS-CoV-2 complex, indicating ADE of infection via FcγRII. No enhancement was observed in parental cells or in B16-F10_ FcγRII incubated with DH1052 and COVA1–16 mAbs. **(B)** Widefield fluorescence microscopy confirmed these results: parental B16-F10 and FcγRII-expressing B16-F10 cells were exposed to SARS-CoV-2_eGFP (MOI 0.04), following preincubation for 1 h with mAb DH1047 (10 μg/mL starting concentration, 3-fold dilution series) for 24 and 48 h. No GFP signal was detected in parental cells, while FcγRII-expressing cells showed strong GFP fluorescence, particularly at 10 μg/mL and 3 μg/mL of DH1047. Images were acquired using a GE Healthcare DeltaVision microscope with a U Plan FI × 20 dry objective. Each image is a nine-panel stitch (1,024 pixels × 1,024 pixels each). Nuclei are shown in blue.

### DH1047 monoclonal antibody facilitates SARS-CoV-2 uptake in hMDMs

We therefore hypothesized that SARS-CoV-2 might enter hMDMs via an alternative, antibody-mediated pathway, involving FcγRs, abundantly expressed on hMDMs (**Supplementary Figure S2B**). SARS-CoV-2_nLuc virus was preincubated at MOIs of 0.4 and 0.04 with nine threefold serial dilutions of the three anti-Spike mAbs, starting from 30 μg/mL before incubation with hMDMs. An increase in RLUs (~ 1 × 10^5^/well) was observed in hMDMs exposed to the DH1047-SARS-CoV-2 complex, mostly when the concentration of mAb DH1047 was within the range of 0.1 to 3 μg/mL (**Figure 4A**). In contrast, no signal was detected in cells exposed to mAbs DH1052 or COVA1–16 preincubated with SARS-CoV-2_nLuc under any condition, indicating that enhancement of infection was specifically mediated by mAb DH1047 in a concentration-dependent manner. To visualize the viral infection, SARS-CoV-2_eGFP was preincubated with three threefold dilutions of each antibody (starting from 3 μg/mL). GFP signal was observed in hMDMs only when the virus was preincubated with mAb DH1047 (**Figure 4B**). No signal of replication was detected in cells where the virus was preincubated with mAbs DH1052 or COVA1–16 (**Supplementary Figure S4**). In addition, most of the hMDMs stained for SARS-CoV-2 NP were also GFP-positive cells, indicating that these cells harbored actively replicating viruses (**Figure 4B**). These findings indicate that while hMDMs are not permissive to SARS-CoV-2 infection (**Figures 2B**, right, **C**), mAb DH1047 can mediate ADE of infection *via* FcγRs, enabling viral entry under specific virus-to-antibody concentration conditions.

**Figure 4:**
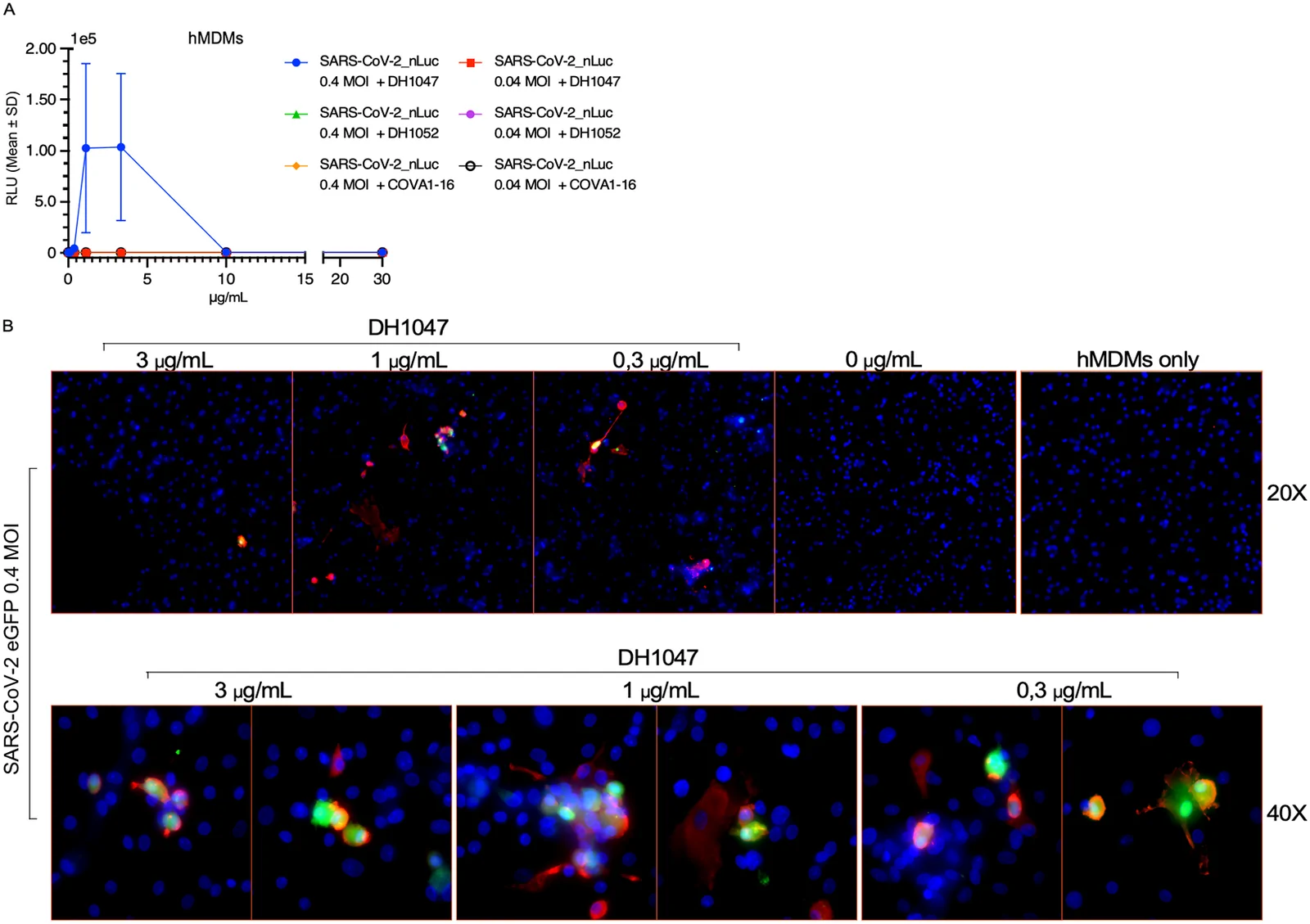
mAb DH1047 enhances SARS-CoV-2 infection in hMDMs. **(A)** hMDMs were infected with SARS-CoV-2-nLuc (MOI 0.4) for 24 h after virus preincubation with threefold serial dilutions of mAbs DH1047, DH1052, or COVA1–16 (starting at 30 µg/mL). A marked increase in RLU was detected when the virus was preincubated with DH1047 (0.1–3 µg/mL), indicating ADE of SARS-CoV-2 infection. **(B)** Widefield fluorescence microscopy confirmed that mAb DH1047 mediates ADE of SARS-CoV-2 infection in hMDMs. SARS-CoV-2_eGFP was pre-incubated for 1 h with mAb DH1047 (3, 1, and 0.1 μg/mL) and added to hMDMs for 24 h. Following incubation, cells were washed, fixed, permeabilized, and stained with a rabbit polyclonal antibody against the SARS-CoV-2 nucleocapsid protein (red). Nuclei were counterstained with Hoechst (blue). Detection of eGFP (green) and SARS-CoV-2 NP indicated viral replication. Images were acquired using a DeltaVision Ultra microscope with a × 20 or × 40 oil-immersion objectives.

### HMDMs support ADE-mediated productive SARS-CoV-2 infection

We next investigated whether SARS-CoV-2 could complete the replication cycle in the infected hMDMs and produce functional new viruses, thus potentially spreading infection in permissive cells. To assess this, SARS-CoV-2_nLuc (MOI 0.4) was preincubated with serial dilutions of mAb DH1047 and subsequently added to hMDMs, which were further cultured for up to 7 days. HMDMs exhibited a mAb dose-dependent increase in luciferase signal when tested at 24 and 48 hpi, with RLU values beginning to rise at a concentration of mAb DH1047 of 0.1 μg/mL and peaking at approximately 1 μg/mL (**Figures 5A, B**), while at day 7 of culture, luciferase activity was nearly undetectable (**Figure 5C**), suggesting transient production of infectious virus. To test the infectivity of the released viruses, the culture supernatant from each experimental condition was transferred to VeroE6 cell cultures for 24 h. Active viral replication was detected only when incubated with supernatant from RLU-positive hMDMs (**Figures 5A–C**, right). These findings confirm that hMDMs, under specific experimental conditions, not only permit viral entry but can support a productive SARS-CoV-2 replication cycle resulting in the release of infectious progeny.

**Figure 5:**
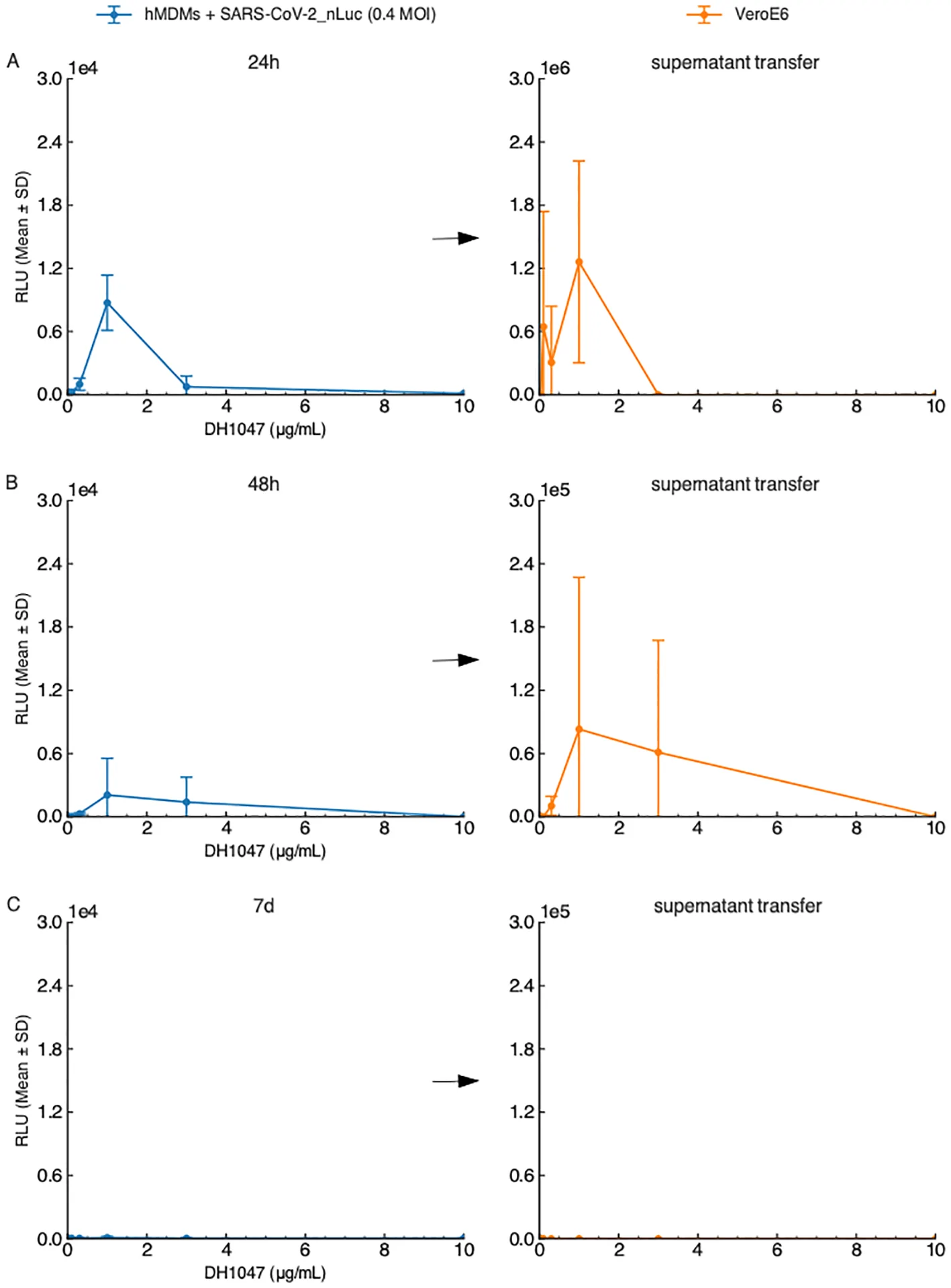
HMDMs support productive SARS-CoV-2 infection via FcγR-mediated entry. HMDMs were exposed to SARS-CoV-2-nLuc for 24 h **(A)**, 48 h **(B)**, and 7 days **(C)** after the virus was preincubated for 1 h with threefold serial dilutions of DH1047 (starting at 10 µg/mL). Supernatants from each well were transferred to VeroE6 cells for an additional 24 h before measuring luciferase activity. **(A)** RLU values in VeroE6 cells were ~ 2 log higher than in hMDMs, indicating that virus produced by hMDMs is replication competent. **(B)** The same assay performed with a 48-h virus-antibody complex on hMDM incubations showed reduced ADE activity compared with that observed at 24 h but still yielded robust luciferase signals in VeroE6 cells, confirming productive infection and secondary viral spread. **(C)** After 7 days, ADE activity had waned in both hMDMs and VeroE6 cells, and luciferase levels returned to baseline.

### Patient’s serum overrides mAb DH1047-driven enhancement of SARS-CoV-2 infection in hMDMs

We hypothesized that severe disease progression in COVID-19 patients could be indicative of SARS-CoV-2 replication enhanced by ADE. We thus selected 13 COVID-19 patients with severe disease progression followed at the I.R.C.C.S. Ospedale San Raffaele during the first pandemic wave, and with serum samples with a known neutralizing antibody (nAbs) titer (**Table 1**) (18). Moreover, we assessed the strength and binding specificity to the full-length SARS-CoV-2 Spike glycoprotein of five sera with the liquid phase luciferase immune precipitation system (LIPS) assay (**Supplementary Table S1**). To determine whether sera from COVID-19 patients could enhance SARS-CoV-2 infection of hMDMs via FcγRs, we first tested the sera at dilutions that included LIPS results comparable to those generated by 1 μg of mAb DH1047, a concentration previously shown to consistently mediate ADE. Despite preincubating SARS-CoV-2_nLuc with a broad range of patients’ serum dilutions, including dilutions corresponding to the ID_50_ neutralization titers and those mirroring the binding activity of mAb DH1047, we did not detect any consistent viral replication in hMDMs (**Supplementary Figure S5A**). Eleven patients’ sera were also tested on the B16-F10_FcγRII cell line for the screening of ADE of SARS-CoV-2 infection (**Supplementary Figure S5B**); however, none showed a consistent enhancement of SARS-CoV-2 infection. These findings suggest that, although these patient sera contain antibodies capable of binding and neutralizing SARS-CoV-2, they do not promote infection enhancement via FcγRs in hMDMs under these experimental conditions. The absence of enhancement observed in two *in vitro* assays, which were shown to be optimal to assess ADE of SARS-CoV-2 infection with mAb DH1047, suggests that factors beyond sera neutralization titers and antigen-antibody binding strength are critical determinants of infection enhancement. We reasoned that the polyclonal mixture of antibodies in patient serum, targeting diverse Spike epitopes, would suppress ADE by outcompeting any minority antibody populations capable of promoting FcγR-mediated enhancement. To further investigate this possibility, we evaluated whether patient sera could modulate mAb DH1047-driven ADE. Coincubation of the mAb with patient serum led to a dose-dependent inhibition of the DH1047-mediated infection until complete abrogation (**Figure 6**). The extent of inhibition showed a trend consistent with the neutralizing potency of the serum (**Table 1**). Interestingly, the serum of a patient with no measurable neutralizing activity and antibody binding (PZ_200471) did not interfere with DH1047-mediated enhancement of SARS-CoV-2 infection, indicating that the inhibition was specifically mediated by antibodies targeting SARS-CoV-2 Spike neutralizing epitopes in the patients’ sera. These findings suggest that the patient’s serum can outcompete mAb for critical epitope binding or FcγR engagement, thereby preventing the enhancement of infection.

**Figure 6:**
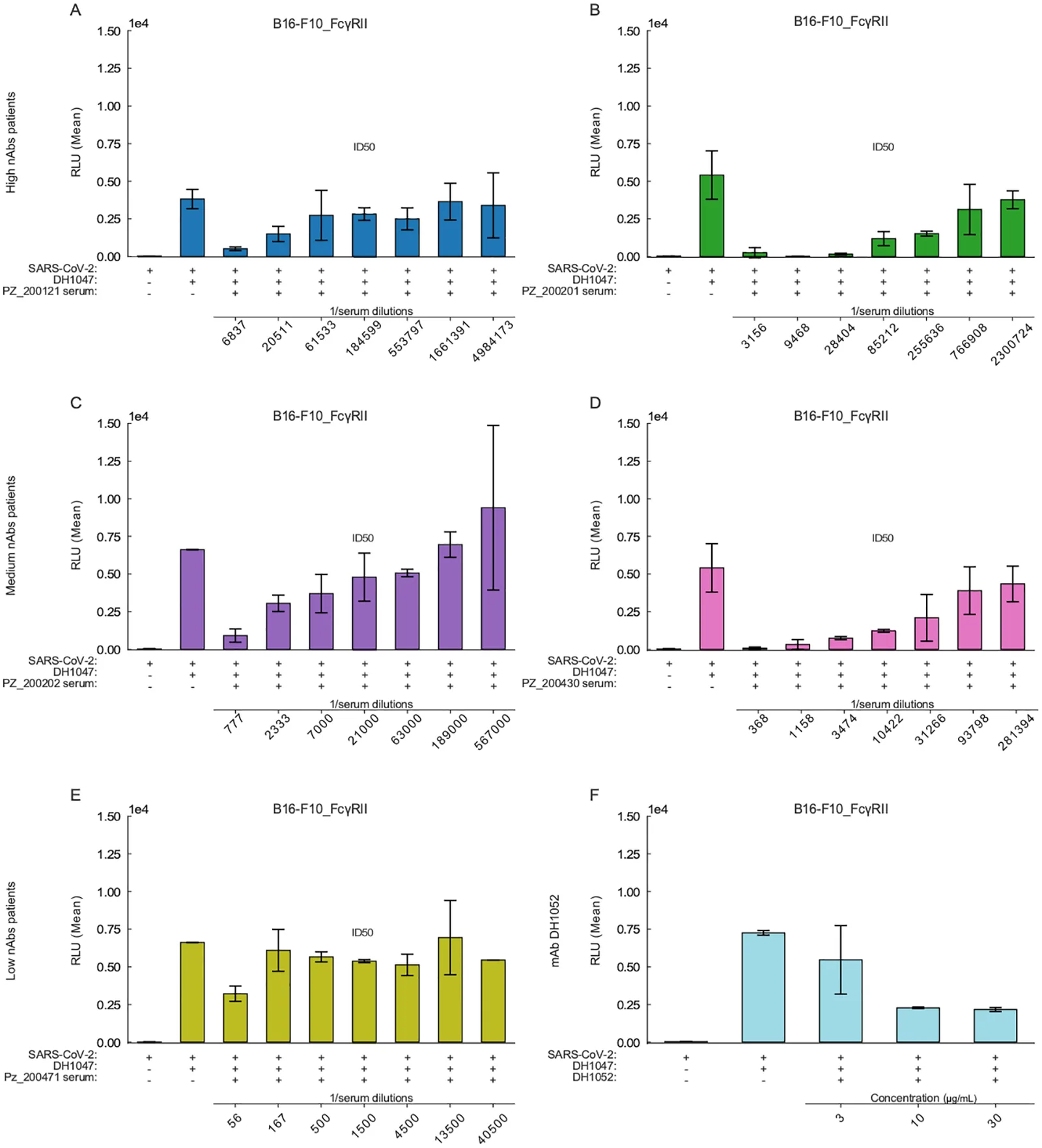
Patient sera inhibit mAb DH1047-mediated enhancement of SARS-CoV-2 infection in B16-F10_FcγRII cells. B16-F10 FcγRII cells were incubated with SARS-CoV-2-nLuc (MOI 0.4) pre-incubated with mAb DH1047 (3 µg/mL) either alone or together with 3-fold serial dilutions of sera from patients PZ_200200 **(A)**, PZ_200201 **(B)**, PZ_200121 **(C)**, PZ_200430 **(D)**, and PZ_200471 **(E)**, or with mAb DH1052 **(F)**. Patient sera and DH1052 consistently inhibited DH1047-mediated enhancement of infection, in line with their respective ID_50_ neutralization titers.

## Discussion

Our results provide insights into how antibodies can influence SARS-CoV-2 uptake and replication in hMDMs, with particular emphasis on the potential for ADE of infection via FcγRs. First, we confirmed that hMDMs, when exposed to SARS-CoV-2 *in vitro*, do not support productive viral replication and thus do not serve as efficient hosts for the virus. This finding aligns with previous *in vitro* studies indicating that macrophages do not become infected with SARS-CoV-2 (7, 28–30), likely due to their limited expression of ACE2 (31), the primary cellular receptor for viral entry. By using Spike-pseudotyped LV, we showed that although viral particles are readily internalized, they are routed to endolysosomal degradation without establishing replication. These results help contextualize previous publications describing the presence of SARS-CoV-2 RNA or proteins within macrophages of different body compartments (28, 29, 32–34), which may reflect phagocytic uptake rather than infection. Analogously, our results with Spike-pseudotyped LV help to interpret the absence of infection and replication by fully infectious recombinant SARS-CoV-2 in hMDMs, further supporting the conclusion that these cells generally do not sustain viral replication. In this report, we demonstrate that the anti-Spike mAb DH1047, but not DH1052 or COVA1–16, significantly enhanced SARS-CoV-2 infection in hMDMs. mAb DH1047, known to enhance infection in an Fc receptor-dependent manner in FcγRs I and II-expressing TZM-bl cell lines (16), led to SARS-CoV-2 productive infection in hMDMs. DH1047 consistently induced ADE and displayed a comparable antibody concentration-dependent response profile across independent experiments, despite some variability in the luciferase signal between hMDM experimental batches. ADE was only observed at a given ratio between the antibody concentration and the viral titer. This ratio-dependent effect highlights that ADE is a finely regulated phenomenon, in which the availability of antibodies and the ability to bind the FcγRs are crucial factors in determining viral entry.

We further confirmed the production of infectious viral particles by replication in VeroE6 cells following the transfer of culture supernatants from the infected hMDMs. The slow decline after the peak replication at 24 hpi suggests that mechanisms such as macrophage-intrinsic antiviral restriction or virus-induced cell death may constrain both the magnitude and duration of DH1047-mediated ADE *in vitro*. Molecular analyses, including RT-qPCR, quantification of genomic and subgenomic viral RNAs, and approaches specifically assessing viral replication intermediates, would complement the assessment of the extent and duration of SARS-CoV-2 replication in hMDMs.

In a similar study, Pickering et al. (17) used mAbs to test ADE of infection in hMDMs and cell lines expressing FcγRs. Despite using different experimental conditions to generate and culture the hMDMs, such as purification methods, composition of the culture medium, and the duration between differentiation and infection experiments, the results are comparable to ours. While our study’s assessment of ADE was limited to one viral strain, the USA-WA1/2020 isolate of SARS-CoV-2, they used several SARS-CoV-2 variants of concern and showed variant-specific variability of ADE among the monoclonals tested. Therefore, the findings of our study may not necessarily apply to newer variants of SARS-CoV-2, which could have different susceptibilities to ADE or immune evasion mechanisms.

When we tested COVID-19 patients’ sera for ADE in hMDMs, we found no enhancement of infection, despite the sera containing varying levels of nAbs. However, we tested a restricted number of patients’ sera, all with severe disease progression. Our hypothesis that severe disease may reflect the presence of ADE may not be justifiable during the first COVID-19 wave in northern Italy. Analogously, García-Nicolás et al. (7) did not find evidence of ADE of SARS-CoV-2 infection in hMDMs in a handful of COVID-19 convalescent patients’ sera, and Pickering et al. (17) described ADE only in one COVID-19 serum when testing a larger number of patients (*n* = 50) of the first COVID-19 wave in the UK and with any type of disease progression. Recently, Thomas et al. (35) found ADE in 6.2% of COVID-19 infection patients’ sera (primarily severe cases) and 5.3% of vaccinated individuals (adenovirus vector and inactivated virus groups) out of 210 sera, though ADE was assessed only in a cell line-based assay. Thus, ADE may be a rare event, regardless of severe COVID-19, or other disease patterns may be more informative. For example, long COVID may represent a clinical context in which persistent immune activation, diminished antibody responses, and low-level viral persistence could favor antibody-dependent mechanisms that may not be captured during acute severe disease. The lack of ADE using the patient’s sera *in vitro* could be attributed to the complexity of the immune response *in vivo*, as our results, as well as those of other groups (17), suggest that ADE of SARS-CoV-2 infection requires a specific antibody-to-virus ratio. In addition, antibodies in the sera may compete for epitope binding on the virus and on FcγRs, preventing the few ADE-mediating antibodies from facilitating viral entry. Indeed, we showed that mAb DH1052 consistently inhibited mAb DH1047-mediated ADE in a dose-dependent manner, as did the patient serum lacking detectable neutralizing activity at the higher concentrations. This is consistent with competition for FcγR engagement, suggesting that FcγR occupancy itself may limit ADE in polyclonal settings. This competitive inhibition suggests that *in vivo* polyclonal antibody responses, comprising mixtures of neutralizing and nonneutralizing antibodies, may buffer antibodies capable of driving ADE. This may explain why, despite billions of SARS-CoV-2 infections worldwide and unprecedented global vaccine deployment, no clinical syndrome attributable to ADE has yet been convincingly documented. The antibody landscape generated by infection or vaccination appears to favor neutralization and immune clearance rather than FcγR-dependent enhancement. In conclusion, our findings show that while SARS-CoV-2 does not naturally replicate in hMDMs, ADE of SARS-CoV-2 through FcγRs can occur with monoclonal antibodies capable of inducing ADE. This mechanism may contribute to viral persistence and spread within the host, and possibly to the exacerbation of the disease.

## Data Availability

The raw data supporting the conclusions of this article will be made available by the authors, without undue reservation.

## Supplementary material

Supplementary Figure 1 Schematic representation of the expression cassettes of the plasmids used in this study. Lentiviral transfer vectors expressing Luciferase (pGAE-LucW) or mCherry (pGAE-mCherry), plasmid expressing cytoplasmic tail (CT)-truncated (delta-21) Spike glycoprotein from the Wuhan-Hu-1 strain (pSpike-C3), plasmid encoding the G glycoprotein from the vesicular stomatitis virus (phCMV-VSV.G), plasmid encoding the SIV-Gag protein fused to GFP and packaging plasmid pADSIV3+. CMV, cytomegalovirus immediate-early promoter; R, repeat element; U5, 5’ untranslated region; U3, 3’ untranslated region; PBS, primer binding site; SD, splice donor site; Ψ, packaging signal; cPPT, central polypurine tract; RRE, Rev response element; dU3, SIN deletion in U3 region of 3’ LTR; WPRE, woodchuck hepatitis virus post-transcriptional regulatory element; pA, SV40 polyA signal; bGHpA, polyA signal from bovine growth hormon.

Supplementary Figure 2 Expression of Fcγ receptors on B16-F10_FcγRII cells and hMDMs. HMDMs and engineered B16-F10_FcγRII cells were stained and analyzed by flow cytometry for CD14, ACE2, CD16, CD32, and CD64 expression. CD14 positive hMDMs showed negligible ACE2 expression, while exhibiting high expression of FcγRI and FcγRII, and moderate expression of FcγRIII. The engineered B16-F10_FcγRII cell line displayed consistent and robust expression of FcγRII.

Supplementary Figure 3 FcγRII blockade abrogates mAb DH1047 mediated SARS-CoV-2_nLuc infection in B16-F10_FcγRII cells. To confirm that mAb DH1047 mediated infection was specifically dependent on FcγRII engagement, B16-F10_FcγRII cells were pre-incubated with 0.4 µg/mL of an anti-CD32 monoclonal antibody before exposure to SARS-CoV-2_nLuc preincubated with mAb DH1047 at 10 or 3 µg/mL, and then added to cells either in the presence or absence of prior FcγRII blockade. Pre-incubation with the anti-CD32 blocking antibody abrogated the NanoLuc signal at both DH1047 concentrations, demonstrating that DH1047-mediated enhancement of SARS-CoV-2_nLuc infection in B16-F10_FcγRII cells is specifically mediated by FcγRII. NanoLuc activity was measured 24 h post-infection and is expressed as relative light units (RLU). Bars represent the mean ± SD of three replicates.

Supplementary Figure 4 mAb DH1047 mediates ADE of SARS-CoV-2 infection in hMDMs. SARS-CoV-2_nLuc was pre-incubated for 1 h with 3-fold serial dilutions of mAbs DH1047, DH1052, and COVA1-160, starting at 3 µg/mL. The virus antibody complexes were then added to hMDMs and incubated for 24 h. After incubation, cells were washed, fixed, permeabilized and stained with a rabbit polyclonal antibody against the SARS-CoV-2 nucleocapsid protein (red). Nuclei were counterstained with Hoechst (blue). Strong SARS-CoV-2_NP signal (red) was detected in hMDMs exposed to SARS-CoV-2 pre-incubated with mAb DH1047, whereas no signal was observed when the virus was preincubated with mAbs DH1052 or COVA1-16.

Supplementary Figure 5 Sera from SARS-CoV-2 patients do not induce antibody-dependent enhancement of SARS-CoV-2 infection in hMDMs or B16-F10FcγRII cell lines. (A) RLU values were measured in hMDMs incubated with SARS-CoV-2-nLuc (MOI 0.4) for 24 h after virus pre-incubation with ten 3-fold serial dilutions of sera from five COVID-19 patients (PZ_200121, PZ_200201, PZ_200202, PZ_200430, and PZ_200471) or with mAb DH1047 (positive control). Patient sera did not enhance infection, whereas DH1047 induced a marked RLU increase. (B) In the B16-F10 FcγRII cell line, none of the 11 patient sera promoted infection, while mAb DH1047 induced clear enhancement, indicating absence of serum-mediated ADE of SARS-CoV-2 infection.

Supplementary Table 1 Binding signals, measured as light units (LU) using a liquid-phase Luciferase Immunoprecipitation System (LIPS) assay, were obtained for serial dilutions of the DH1047 monoclonal antibody starting at 10 μg/mL, and for patient sera. These signals were compared to identify serum dilutions that exhibited binding levels comparable to 1 μg of DH1047.
